# *WSB1* and *IL21R* Genetic Variants Are Involved in Th2 Immune Responses to *Ascaris lumbricoides*

**DOI:** 10.3389/fimmu.2021.622051

**Published:** 2021-02-22

**Authors:** Valdirene Leão Carneiro, Hugo Bernardino Ferreira da Silva, Gerson de Almeida Queiroz, Rafael Valente Veiga, Pablo Rafael Silveira Oliveira, Norma Vilany Queiroz Carneiro, Anaque de Oliveira Pires, Raimon Rios da Silva, Flavia Sena, Emilia Belitardo, Regina Nascimento, Milca Silva, Cintia Rodrigues Marques, Ryan dos Santos Costa, Neuza Maria Alcantra-Neves, Mauricio L. Barreto, Philip J. Cooper, Camila Alexandrina Figueiredo

**Affiliations:** ^1^Department of Life Sciences, State University of Bahia, Salvador, Brazil; ^2^Institute of Health Sciences, Federal University of Bahia, Salvador, Brazil; ^3^Center for Data and Knowledge Integration for Health, Fiocruz, Salvador, Brazil; ^4^Institute of Biological Sciences, Federal University of Bahia, Salvador, Brazil; ^5^Multidisciplinary Health Institute, Federal University of Bahia, Salvador, Brazil; ^6^Institute of Collective Health, Federal University of Bahia, Salvador, Brazil; ^7^School of Medicine, International University of Ecuador, Quito, Ecuador; ^8^St. George's University of London, London, United Kingdom

**Keywords:** *Ascaris lumbricoides*, immunity, polymorphism, *WSB1*, *IL21R*

## Abstract

Genetic and epigenetic factors are considered to be critical for host-parasite interactions. There are limited data on the role of such factors during human infections with *Ascaris lumbricoides*. Here, we describe the potential role of genetic factors as determinants of the Th2 immune response to *A. lumbricoides* in Brazilian children. Stool samples were collected from the children to detect *A. lumbricoides* by microscopy and peripheral blood leukocytes (PBLs) were cultured in whole blood cultures for detection of cytokines (IL-5, IL-10, and IL-13) *in vitro*. Levels of anti-*A. lumbricoides* IgE and IgG4 were measured in plasma. DNA was extracted from PBLs and genotyped using Illumina 2.5 Human Omni Beadchip. Candidate genes associated with *A. lumbricoides* responses were identified and SNVs in these selected genes associated with the Th2 immune response to *A. lumbricoides*. Haplotype, gene expression, and epigenetic analyses were done to identify potential associations with Th2 immune responses. GWAS on samples from 1,189 children identified *WSB1* as a candidate gene, and IL-21R was selected as a biologically relevant linked gene for further analysis. Variants in *WSB1* and *IL21R* were associated with markers of Th2 immune responses: increased *A. lumbricoides*-specific IgE and IL-5/IL-13 by PBLs from infected compared to uninfected individuals. In infected children, *WSB1* but not *IL21R* gene expression was suppressed and increased methylation was observed in the *WSB1* promoter region. This is the first study to show an association between genetic variants in *WSB1* and *IL21R* and Th2 immune responses during *A. lumbricoides* infections in children. *WSB1*/*IL21R* pathways could provide a potential target for the treatment of Th2-mediated diseases.

## Introduction

A quarter of the world's population is estimated to be infected with soil-transmitted helminth (STH) parasites. The highest prevalence occurs among children living in rural areas of the tropics in conditions of poverty with limited access to treated water and sanitation (1, 2). Among STH infections, *Ascaris lumbricoides* infection is estimated to infect 820 millions causing a significant burden of morbidity and mortality, the latter generally being associated with intestinal obstruction (3–5). Chronic infections in children, particularly among those with high parasite burdens, can impair host nutrition leading to growth stunting and diminished cognitive development (1, 6).

*Ascaris lumbricoides* infection induces strong Th2-type immune responses in infected humans leading to the production of high circulating levels of total and parasite-specific IgE, generally targeted at larvae that undergo a phase of extra-intestinal migration through the lungs. Th2-induced host protective mechanisms against *A. lumbricoides* parasites include eosinophil-mediated killing of larvae in the tissues, mast-cell degranulation in the tissues and intestinal tract, and increased intestinal mucus production through goblet-cell hyperplasia (7, 8).

*WSB1, IL21*, and *IL21R* genes are important regulators of the IgE response. The *WSB1* gene has a role in the regulation and maturation of the interleukin-21 receptor (IL-21R) (9). The *WSB1* gene was initially described through its relationship with the suppressor-protein-signaling box (SOCS) cytokine family (10, 11). The *IL21R* gene is constitutively expressed on T and B lymphocytes and NK cells (12) and has effects that vary according to the stage of cell differentiation. B cell proliferation and differentiation into plasma cell *in vitro* appear to occur via IL-21 signaling (13) and IL-21R knock-out mice have high levels of IgE and reduced IgG1. In mice, IL-21 inhibits IgE responses through the IL-21 receptor on B cells, triggering IL-4-independent signaling of STAT3 (14). IL21/*IL21R* binding activates STAT-3 and production of interferon-gamma by T cells and NK cells that counteracts the effects of IL-4 on IgE production (13). In contrast, IL-21 activates STAT-3 in human B cells and acts synergistically with IL-4 to increase the secretion of IgE (14). Other studies in humans have shown that IL-21 can suppress IgE synthesis, indicating that effects of the IL-21/IL-12R pathway on IgE production may be affected by host genetics: genetic variants in the *IL21R* gene associated with IgE production have been identified by GWAS (15, 16). IL-21R may have a critical role in the control of allergic responses and helminth infections (17, 18).

The host immune response, during the course of a helminth infection such as *A. lumbricoides*, involves the induction of complex immune responses that include protective Th2-mediated protective mechanisms. Host genetics is likely to play a key role in resistance and susceptibility to *A. lumbricoides* (19). Loci shown to be associated with susceptibility to helminth infection include 5q31-q33, signal transducer and transcriptional activator 6 (STAT6) and ligase 4 (LIG4) (20–22). To date, no genome-wide association studies have addressed the role of host genetics in Th2 responses to *A. lumbricoides* infection and limited candidate-genes studies have been done (22–24). Recently, positive associations between epigenetic alterations of increased histone acetylation and type 2 immune responses including IgE have been observed among individuals infected with *A. lumbricoides* (25).

In the present study, we used a variety of strategies to study genetic determinants of the host Th2 immune response during *A. lumbricoides* infection in children that included gene discovery using a genome-wide approach and a candidate gene approach based on the findings of the former. This was followed by expression quantitative trait loci and epigenetic analyses to explore how genetic variations in candidate genes are linked to host Th2 immune response during *A. lumbricoides* infection.

## Methods

### Characterization of the Reference Population

This study was done among children and adolescents in the city of Salvador, Brazil, that has a population of 2.8 millions. The study sample has been described in detail elsewhere (2, 26, 27). Briefly, 1,445 children were recruited in early childhood into a prospective study to measure the impact of a sanitation program in the city of Salvador on child morbidity (28). Data were collected from children born between 1994 and 2001, who lived in sentinel areas of the city. Standardized questionnaires were applied to the legal guardian of each child between 1997 and 2003 (baseline) to collect data on demographic and social variables, as well as on the domestic environment. In 2000, fecal samples were collected for detection of geohelminth parasites by microscopy. The children were surveyed again in 2005 to obtain stool and blood samples for laboratory tests and extraction of genomic DNA.

### Ethics

The Brazilian National Research Ethics Committee approved the study protocol and informed written consent was obtained from the legal guardian of each child/adolescent (Resolution Number: 15895).

### Blood Collection and Cell Culture

Blood samples were collected in heparinized tubes and peripheral blood leukocytes (PBLs) were cultured in whole blood at a dilution of 1:4 in RPMI medium (Gibco, Auckland, New Zealand), supplemented with 10 mmol/L glutamine (Sigma-Aldrich, St Louis, USA) and 100 μg/ml gentamicin (Sigma-Aldrich, St Louis, USA). PBLs were cultured within 6 h of collection in the presence of *A. lumbricoides* antigen (10 ug/mL, endotoxin-free), pokeweed mitogen (2.5 ug/mL), or no stimulant, in a humidified environment at 37°C with 5% CO_2_ for 5 days. Supernatant fluids were harvested for 24 h (IL-10) or 5 days of cultures (IL-5 and IL-13) (2, 29).

### IL-10, IL-13, and IL-5 Measurements

Concentrations of IL-5, IL-10, and IL-13 in cell culture supernatant were measured using commercial sandwich ELISAs following the manufacturer's instructions (BD PharMingen, San Diego, CA, USA). Cytokine concentrations were dichotomized into responders and non-responders using the lowest detection level for each cytokine. Low/high detection limits (in pg/ml) were 15.6/500 for IL-5, 62.5/4,000 for IL-13 and 31.25/500 for IL-10. The number of individuals evaluated for IL-5 and IL-13 production were 67 and 73, respectively.

### Parasitological Analysis

Two fecal samples were collected from each child, separated by a 2-week interval, and analyzed for *A. lumbricoides* infection using sedimentation (30) and Kato-Katz methods (31) as described (21). Positive children were defined by the presence of *A. lumbricoides* eggs detected by either method. All positive children were treated with appropriate anthelmintics (26).

### IgE and IgG4 Anti-*A. lumbricoides* Antibodies Serum Concentrations

The ImmunoCAP assay (Phadia Diagnostics AB, Uppsala, Sweden) was used for determination of specific IgE serum concentrations against *Ascaris* and positive samples had ≥0.35 kU/L of anti–*A. lumbricoides* IgE. Anti–*A. lumbricoides* IgG4 was detected using an indirect ELISA as described previously (32).

### Genotyping and Quality Control

Genotyping was performed using the Illumina BeadChip Human Omni2.5-8 Kit (www.illumina.com), by the Consortium EPIGEN-Brazil (https://epigen.grude.ufmg.br/). One individual was excluded due to inconsistency between registered and genetic sex, based on X chromosome SNVs and 61 were removed based on kinship coefficients (≥0.1, to include second-degree relatives) between pairs of individuals (33). SNVs excluded from the analysis were: on X, Y and mitochondrial chromosomes; genotyping call rate <0.98; and deviance in the Hardy-Weinberg equilibrium with a *P*-value <10^−4^ and Minor Allele Frequency (MAF) <1% (34). After quality control, 1,857,191 autosomal SNVs were included. A total of 636 individuals had detectable values of IgE and/or IgG4 and were included in the analysis. Linear regression was done using ln-transformed ratio of anti-*A. lumbricoides* IgE to IgG4 (35). Through this genome-wide analysis, we selected SNVs in the *WSB1* gene pathway for a candidate gene approach based on biological role in immune response from among the top 20 hits. The closely linked gene pathway for *IL21R* was selected also. Genotype information for these two genes was extracted from the chip at the following regions: *WSB1* from 27294080 to 27315926 (location: NC_000017.11) position at chromosome 17. *IL21R* from 27402162 to 27452043 (location: NC_000016.10) position at chromosome, 16 and a candidate-gene analysis was done for both genes. For quality control, the following filters were applied: genotyping call rate >90%, imbalance of Hardy-Weinberg equilibrium with *P* < 0.05 and the Minor Allele Frequency (MAF) >1% (34). A total of 12 markers on *WSB1* and 35 markers on *IL21R* were analyzed after quality control. These data are deposited in the European Nucleotide Archive [PRJEB9080 (ERP010139) Genomic Epidemiology of Complex Diseases in Population-Based Brazilian Cohorts], Accession No. EGAS00001001245, under EPIGEN Committee Controlled Access mode.

### Real-Time Quantitative Polymerase Chain Reaction (qRT-PCR)

To evaluate the expression levels of *WSB1* and *IL21R* genes, RNA was isolated from PBL cultures using RNeasy Mini Kit (Qiagen, Hamburg, Germany) and 0.3 μg of total RNA from each sample was reverse transcribed into cDNA using 200 U of Superscript III Reverse Transcriptase (Life Technologies, Carlsbad, CA, USA) and 500 ng of Oligo (dT) (Life Technologies, Carlsbad, CA, USA), as described previously (36). Pre-synthesized Taqman® Gene Expression Assays (Applied Biosystems, Foster City, CA, USA) were used to amplify *WSB1* (Hs00373204_m1), *IL21R* (Hs00222310_m1) and β*-actin* (Hs01060665_g1). cDNA was detected using QuantStudio 12K Sequence Detection System (Applied Biosystems, Foster City, CA, USA). Each qRT-PCR assay was performed with 10 ng of cDNA in 10 μL of Taqman-PCR Master mix 2X (Applied Biosystems, Foster City, CA, USA) and 1 μL of primer/probe set and purified using deionized H_2_O q.s. 20 μL. Gene expression was normalized to β*-actin* levels. Relative quantification was performed using the comparative threshold cycle (ΔΔCT) method (37–39).

### *In silico* Functional Analysis

RegulomeDB (regulomedb.org) is a database for interpretation of regulatory variants in the human genome. It includes high-throughput, experimental datasets from ENCODE (Encyclopedia of DNA Elements) and other sources. A score ranging from 1 to 6 is attributed for each SNV; the lower the score, the greater the presumed involvement in regulatory processes (40).

### DNA Methylation Assessment

We used an epigenetic approach to determine the level of methylation on the promoter region *WSB1* following infection with *A. lumbricoides* using OneStep qMethyl kit (Zymo Research). Primers within the CpG rich (promoter) region of *WSB1* were: forward, 5′-CAG GCC TTT GCA ATG TTT AGG-3′; reverse, 5′-AGC CAG CAG GTT TTA GGA AGG-3′. Methylation percentages were obtained using 20 ng of DNA in duplicate in test and reference reaction mixes. Reactions were done using a QuantStudio 12K Sequence Detection System (Applied Biosystems, Foster City, CA, USA) as follows: 2 h 37°C; 10 min 95°C; 40 cycles 30 s 95°C, 1 min 54°C, 1 min 72°C followed by an dissociation stage to check specificity of PCR products. The Ct values obtained were used to calculate ΔCt values Ct (test) and Ct (reference). Methylation percentages were calculated as the product of 100 × 2–ΔCt.

### Statistical Analysis

For GWAS, linear regressions were done to evaluate the association between SNVs and lg (anti-*A. lumbricoides* IgE/ anti-*A. lumbricoides* IgG4) using additive models. Power for genetic association analyses depends on effects of individual polymorphisms (depending on both allelic frequency and associated OR/beta), sample size, and type I error. In the context of GWAS, it is common to consider two levels of significance: a more stringent level such as 5 × 10^−8^ which may allow a conclusion of statistical significance, and a less stringent level such as 1 × 10^−5^ to identify potentially suggestive associations. Using an additive model and a type I error of 1 × 10^−5^, our sample of 996 uninfected and 189 infected children had a power of 80% to detect a polymorphism with beta of 0.3 and frequency >0.15. The statistical power calculation was done using Quanto software (v1.2.4). Models were controlled for confounding by population stratification by inclusion of the first three components of a principal components analysis (PCA) of ancestry informative markers (AIMs) as described (41). In addition, the genomic inflation factor (λ) was estimated to visualize and avoid inflated test statistics (42). Quantile-quantile (Q-Q) plots were used to evaluate the overall significance of the genome-wide association results (Supplementary Figure 2). Associations between polymorphisms in *WSB1* or *IL21R* and *A. lumbricoides* infection, and IL-5, IL-13, and IL-10 cytokine production by PBLs stimulated with *A. lumbricoides* and anti-*A. lumbricoides* IgE and IgG4 were done using logistic regression model in which multivariate models were adjusted for sex, age, and ancestry (first 2 components of PCA analysis of AIMs). Principal components (PC1 and PC2) have categorized individuals according to their ethnic characteristics. Additive models were used in all analyses. Adaptive permutations were also done in adjusted and unadjusted analyses. A computationally intensive procedure based on 1,000,000 permutations was used to estimate the statistical significance of multiple correlation tests in the genetic association analysis (43). Haplotype and genetic risk score analysis were performed using SNPStats program (https://www.snpstats.net/start.htm) (44). Linkage disequilibrium (LD) analysis was done for selected SNVs. Haploview 4.2 software was used to calculate the degree of confidence in the *R*^2^-value. Mann-Whitney or Kruskal-Wallis-tests were used to compare continuous variables and the Chi-squared-test to compare frequencies of categorical variables. Except as specified for GWAS, statistical significance was inferred by *P* < 0.05. Statistical analyses were done using PLINK 1.9 software (www.cog-genomics.org/plink/1.9/), R Statistical Software (Foundation for Statistical Computing, Vienna, Austria), and Prism software version 6 (GraphPad Inc., San Diego, CA).

## Results

### Characteristics of the Study Population

Of 1,246 children eligible, 61 did not have stool data for *A. lumbricoides* infection and were excluded from the analysis, leaving 1,189 (996 non-infected and 189 *A. lumbricoides* infected) children with complete data. Baseline characteristics of the analysis sample are shown in Table 1. Levels of anti-*A. lumbricoides* IgE and IgG4, and levels of IL-5 produced by *A. lumbricoides*-stimulated PBLs were greater among infected than non-infected children (*P* < 0.001).

**Table 1:** Baseline characteristics and immunological markers of *A. lumbricoides* infection among 1,189 children, stratified by *A. lumbricoides* infection.

	**Subject group**				
**Variables**	**Infected** **(*N*)**	**%**	**Uninfected** **(*N*)**	**%**	*P*-value^*^
	189	15.9	996	84.1	
**Sex**					
Male	106	56.1	537	53.7	0.558
Female	83	43.9	459	45.9	
**Age**					
≤ 5	60	31.7	379	37.9	0.133
6–7	64	33.9	348	34.8	
≥8	65	34.4	269	26.9	
**Anti-*****A. lumbricoides***					
IgE	134	70.9	458	45.8	<0.001
IgG4	65	34.4	126	12.6	<0.001
**Cytokine production by** ***A. lumbricoides*****-stimulated peripheral blood cells**^**#**^					
IL-5	36	19.0	95	9.5	<0.001
IL-13	41	21.7	185	18.5	0.589
IL-10	13	6.9	36	3.6	0.082

* P-values were derived using the chi-squared-test.

# *Percentage of responders children for each cytokine evaluated. Responders were defined as those children with cytokine concentrations above the lower detection limits for IL-5 (>15.63 pg/mL), IL-13 (>62.5 pg/mL), and IL-10 (>31.25 pg/mL)*.

### Genome Wide Association Study for SNVs Linked to Parasite-Specific IgE/IgG4 Responses

**Figure 1:**
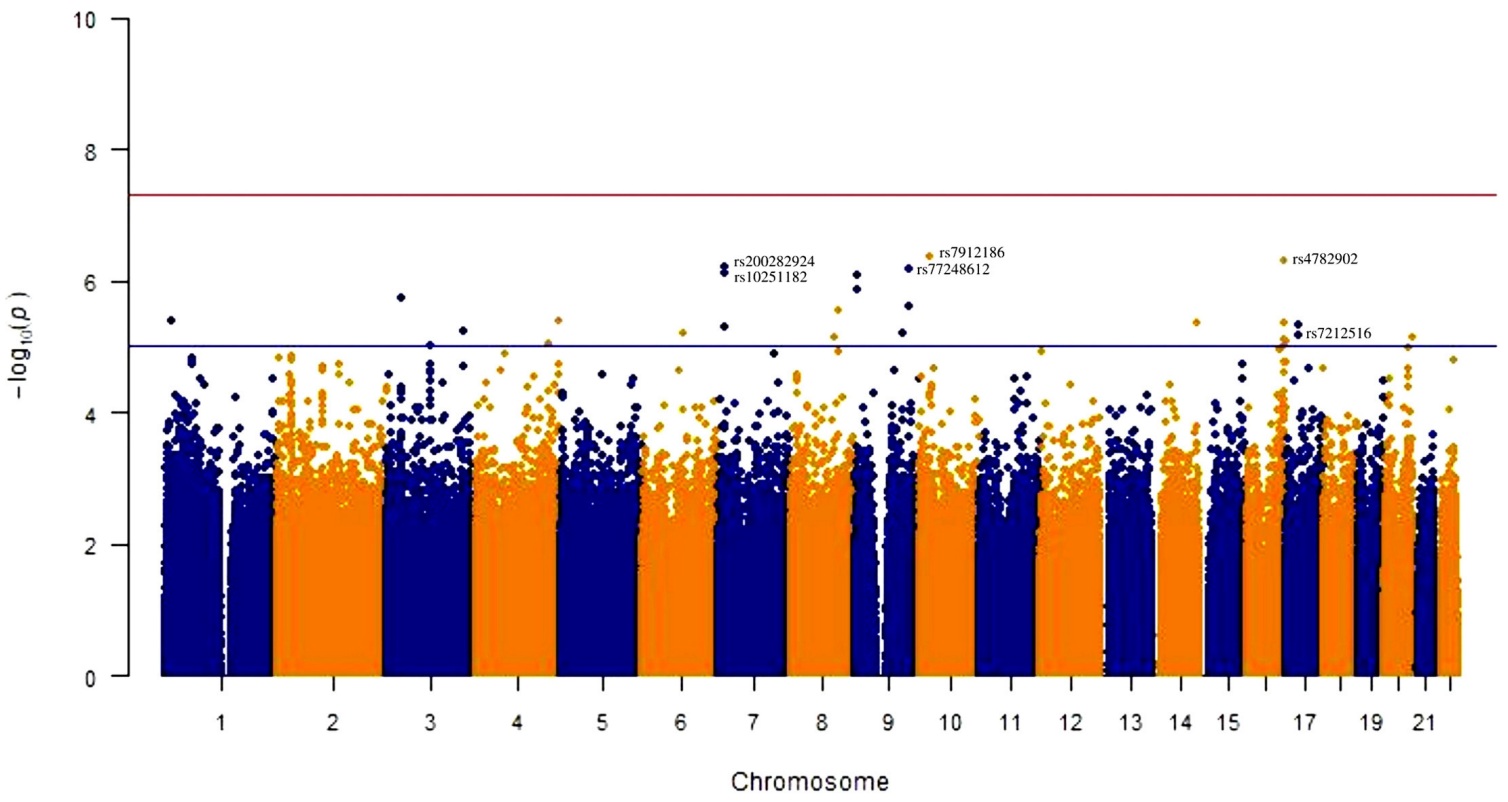
Manhattan plot for genome-wide association with anti-*A. lumbricoide* IgE/IgG4 ratio for 636 subjects. Each single nucleotide variants (SNVs) was tested for association by linear regression using an additive regression model, adjusted for ancestry markers. The red line indicates genome-wide significance (*p* = 5 × 10^−8^) and blue line suggestive significance (*P* = 1 × 10^−5^).

The Manhattan plot for the genome wide analysis of SNVs associated with anti-*A. lumbricoides* IgE/IgG4 are shown in Figure 1 and the top 20 SNVs identified are listed in Table 2 with results of a mapping analysis for these provided in Supplementary Table 1. Among identified SNVs, rs7212516 (Beta: 0.33, CI: 0.18–0.47, *P* = 6.675 × 10^−06^), is an intronic variant located in the *WSB1* (WD repeat and SOCS box containing 1) gene and plays an important role in IgE production (9). Further analyses were focused on *WSB1* and the linked gene, *IL21R*. In addition, SNV rs3093406 in *IL21R* was significantly associated with IgE/IgG4 ratio in GWAS (*P* = 0.0222).

**Table 2:** The results for the 20 best hits in the genome wide association with study anti-*A. lumbricoide* IgE/IgG4.

**Rank**	**Chr**	**SNV**	**Position (bp)^**#**^**	**Risk allele**	**MAF**	**Gene**	**β (CI 95%)**	***p***
1	10	rs7912186	25602870	C	0.02	GPR158	0.75 (0.46–1.04)	4.176 × 10^−07^
2	16	rs4782902	82449332	C	0.47	Intergenic	0.25 (0.15–0.35)	4.89 × 10^−07^
3	7	rs200282924	12171197	C	0.46	Intergenic	0.23 (0.14–0.33)	6.341 × 10^−07^
4	9	rs77248612	120566205	A	0.02	LOC105376244	0.80 (0.49–1.12)	6.896 × 10^−07^
5	7	rs10251182	12171373	T	0.46	Intergenic	0.23 (0.14–0.33)	7.527 × 10^−07^
6	9	rs10081726	2021814	T	0.08	SMARCA2	0.40 (0.24–0.56)	8.095 × 10^−07^
7	9	rs12550848	2021655	T	0.08	SMARCA2	0.39 (0.23–0.55)	1.379 × 10^−06^
8	3	rs4645161	31977744	T	0.45	OSBPL10	0.23 (0.14–0.33)	1.795 × 10^−06^
9	9	rs77772209	120537157	A	0.02	Intergenic	0.73 (0.46–1.12)	2.464 × 10^−06^
10	8	rs77284244	108679300	A	0.03	Intergenic	0.71 (0.41–1.00)	2.771 × 10^−06^
11	1	rs12738424	1.5E+07	G	0.42	KAZN	0.23 (0.13–0.33)	4.084 × 10^−06^
12	4	rs7653904	186607977	T	0.35	SORBS2	0.23 (0.13–0.33)	4.136 × 10^−06^
13	16	rs1025065	82451159	T	0.46	Intergenic	0.23 (0.13–0.33)	4.37 × 10^−06^
14	14	rs61992474	102532899	C	0.13	Intergenic	0.30 (0.43–0.17)	4.377 × 10^−06^
15	17	rs7219758	25597243	G	0.13	Intergenic	0.34 (0.19–0.48)	4.91 × 10^−06^
16	7	rs11980827	12171659	C	0.47	Intergenic	0.21 (0.12–0.31)	5.221 × 10^−06^
17	3	rs6444926	170133235	T	0.12	Intergenic	0.30 (0.17–0.44)	5.788 × 10^−06^
18	6	rs1998219	92382995	T	0.08	CASC6	0.41 (0.23–0.59)	6.252 × 10^−06^
19	9	rs7018777	104794048	G	0.02	Intergenic	0.72 (0.40–1.03)	6.493 × 10^−06^
20	17	rs7212516	25621797	C	0.13	WSB1	0.33 (0.18–0.47)	6.675 × 10^−06^

Analyses were corrected for genetic ancestry.

# *Genomic version GRCh37–hg19; Chr, chromosome; SNV, single nucleotide variation; MAF, Minor Allele Frequency; p, P-value*.

Using log-transformed anti-*A. lumbricoides* IgE and IgG4 as continuous variables: IgG4 was associated with immune response genes such as PRKCA (a kinase that participates in macrophage differentiation induced by macrophage colony-stimulating factor) (*P*-values ranging 10^−5^ and 10^−6^*)*; and continuous IgE was associated with several genes including NKAI2, a sodium-potassium transporter ATPase in T cells (*P*-values ranging 10^−5^ to 10^−6^).

### Associations Between *WSB1* and *IL21R* Variants and Parameters of Host Immune Response to *A. lumbricoides*

The associations between 12 variants in *WSB1* and infection with *A. lumbricoides* and the host immune response to the parasite were studied (Table 3). *P-*values refer to the permutational test. With respect to levels of anti-*A. lumbricoides* specific IgE, SNVs rs7213148, and rs8065359 were positively associated, while rs1060618 and rs9867 were negatively associated. rs9867 was associated with lower production of IL-5 in *A. lumbricoides* antigen-stimulated PBL cultures. With respect to levels of anti-*A. lumbricoides* specific IgG4, rs6505199 and rs9303634 were inversely associated (rs6505199 and rs9303634 are in total LD- see Supplementary Figure 1A) and rs7212516 was positively associated.

**Table 3:** Significant associations between SNVs on *WSB1* and parameters of the host immune response to *A. lumbricoides* including specific IgE and IgG4, and IL-5 production by *A. lumbricoides* antigen-stimulated PBLs.

**SNV**	**MAF**	A1^*^	**Model**	**OR**	**CI 95%**	***P*-value**
**Anti-*****Ascaris lumbricoides*** **IgE**						
rs7213148	0.02	T	ADD	1.98	1.16–3.36	0.009
rs8065359	0.09	A	ADD	1.47	1.10–1.96	0.016
rs1060618	0.36	G	ADD	0.79	0.66–0.93	0.005
rs9867	0.03	A	ADD	0.67	0.43–0.98	0.027
**Anti-*****Ascaris lumbricoides*** **IgG4**						
rs6505199	0.43	G	ADD	0.78	0.63–0.97	0.038
rs9303634	0.43	T	ADD	0.78	0.63–0.97	0.038
rs7212516	0.13	T	ADD	1.39	1.01–1.90	0.034
**IL-5 production in** ***Ascaris lumbricoides*****-stimulated blood cell cultures**						
rs9867	0.03	A	ADD	0.40	0.16–0.99	0.047

* *A1, minor allele; SNV, single nucleotide variation; MAF, Minor Allele Frequency; OR, Odds ratio; P-value, permutacional-test*.

Because *WSB1* is functionally related to IL-21R activation, we scanned the *IL21R* gene for SNVs associated to *A. lumbricoides*. Table 4 shows significant associations between *IL21R* polymorphisms with the presence of measured parameters of infection and immune response to *A. lumbricoides*. The SNVs rs9938401 and rs3093406 were positively associated with a presence of active *A. lumbricoides* infection. SNVs rs3093412 and rs179763 were inversely associated with levels of anti-*A. lumbricoides* IgG4. Four SNVs were inversely associated with levels of anti *A. lumbricoides* IgE (T allele rs76678990; T allele rs58579343; A allele rs11074859; and T allele rs4140673), while two SNVs were positively associated (T allele rs3093308; A allele, rs115350516). These latter two SNVs were associated also with higher IL-5 production by PBLs stimulated with parasite antigens (T allele, rs3093308; A allele, rs115350516) as were rs3093319 and rs77718993. The SNV rs3091236 was positively associated with IL-10 production while rs9930086 was negatively associated. A high degree of linkage disequilibrium was seen between rs58579343 and rs11074859 (see Supplementary Figure 1B).

**Table 4:** Significant associations between SNVs on *IL21R* and infection with *A. lumbricoides* and levels of anti-*Ascaris lumbricoides* IgE and IgG4 and parasite antigen induced production of IL-5 and IL-10 by PBLs.

**SNV**	**MAF**	**A1**	**Model**	**OR**	**CI 95%**	***P*-value**
**Ascaris infection**						
rs9938401	0.48	A	ADD	1.34	1.07–1.96	0.012
rs3093406	0.36	T	ADD	1.82	1.12–2.96	0.018
**Anti-*****Ascaris lumbricoides*** **IgE**						
rs76678990	0.08	T	ADD	0.65	0.48–0.88	0.004
rs58579343	0.21	T	ADD	0.77	0.63–0.95	0.016
rs11074859	0.18	A	ADD	0.77	0.62–0.95	0.017
rs4140673	0.30	T	ADD	0.82	0.69–0.99	0.033
rs115350516	0.23	A	ADD	1.75	1.08–2.83	0.027
**Anti-*****Ascaris lumbricoides*** **IgG4**						
rs3093412	0.33	T	ADD	0.25	0.10–0.61	0.002
rs179763	0.23	C	ADD	0.73	0.56–0.97	0.029
**IL-10 production in** ***Ascaris lumbricoides*****-stimulated blood cell cultures**						
rs3091236	0.21	T	ADD	1.78	1.11–2.85	0.017
rs9930086	0.36	C	ADD	0.55	0.34–0.89	0.019
**IL-5 production in** ***Ascaris lumbricoides*****-stimulated blood cell cultures**						
rs3093319	0.10	G	ADD	1.40	1.05–1.87	0.031
rs115350516	0.03	A	ADD	2.06	1.11–3.83	0.017
rs3093308	0.21	T	ADD	1.40	1.03–1.89	0.025
rs77718993	0.02	T	ADD	1.93	1.01–3.68	0.037

*A1, minor allele; SNV, single nucleotide variation; MAF, Minor Allele Frequency; OD, Odds ratio; P-value, Permutacional-test*.

Haplotype analysis for *IL21R* SNVs rs115350516 and rs3093308 showed that PBLs from individuals with haplotypes GT and AC produced greater levels of IL-5 when stimulated with parasite antigen (Table 5). Individuals with haplotype AC produced greater levels of anti-*A. lumbricoides* IgE.

**Table 5:** Associations between haplotypes for rs115350516 and rs3093308 in the *IL21R* gene and levels of anti-*A. lumbricoides* IgE and IL-5 produced by *A. lumbricoides*-stimulated PBLs.

**Haplotype**	**rs115350516**	**rs3093308**	**Frequency**	**OR^**a**^ (95% CI)**	***P*-value**
**IL-5 production in** ***A. lumbricoides*****-stimulated PBLs**					
**Reference**	G	C	0.74	1	—
Haplotype1	G	T	0.23	1.73 (1.19–2.52)	0.004
Haplotype2	A	C	0.03	3.31 (1.60–6.88)	0.001
**Anti-*****A. lumbricoides*** **IgE**					
Haplotype1	A	C	0.03	2.08 (1.14–3.80)	0.018

a *Adjusted for gender, age, and ancestry markers; OD, Odds ratio*.

### SNV rs3093308 in *IL21R* Is Associated With Type 2 Cytokine Production

**Figure 2:**
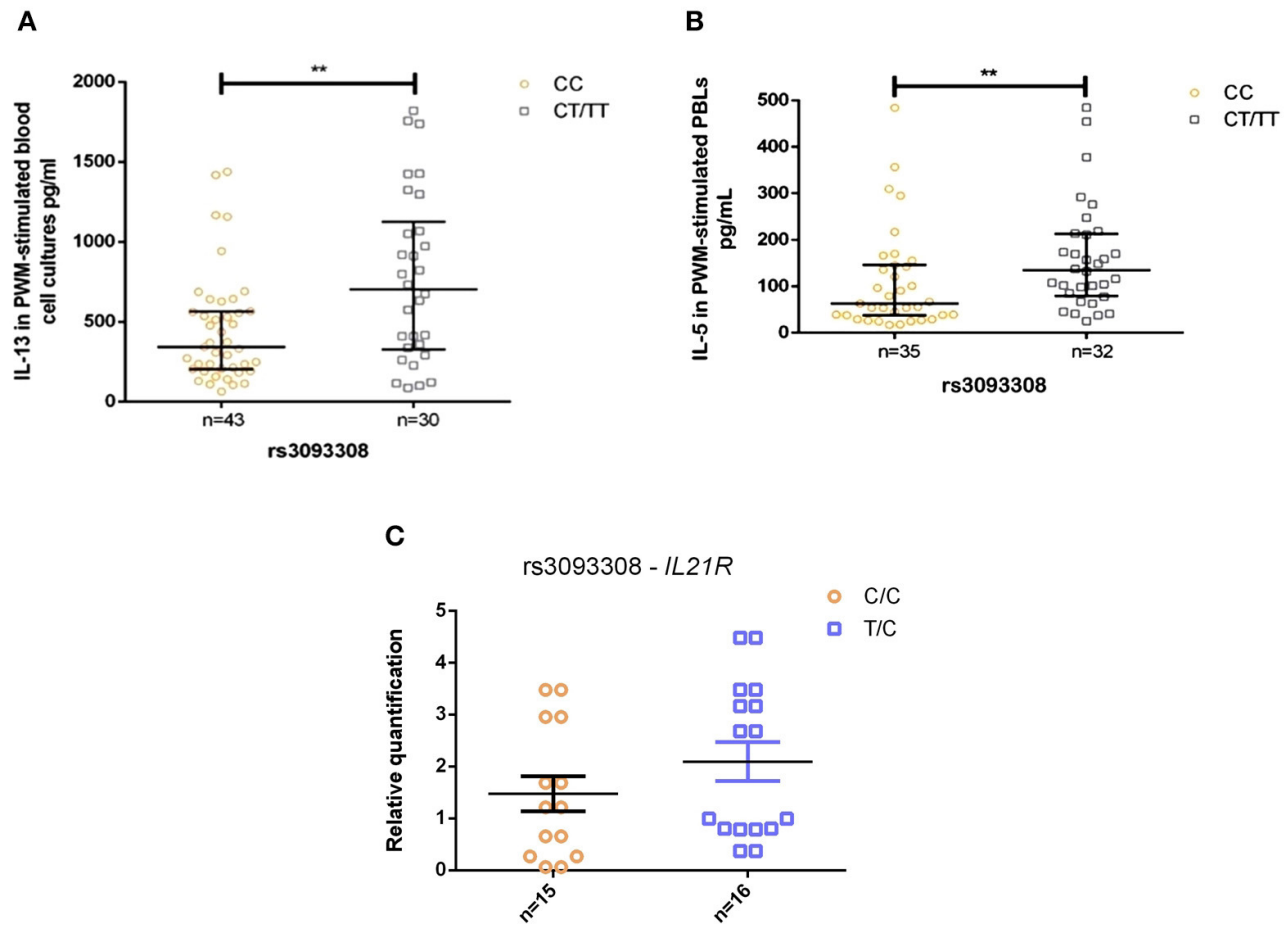
Levels (pg/ml) of IL-5 and IL-13 in *A. lumbricoides* infected subjects produced by parasite antigen stimulated PBLs, by allele for SNV rs3093308 in *IL21R*: **(A)** IL-13, **(B)** IL-5, and **(C)** gene expression analysis for *IL21R* in PBLs (non-significant). CC, genotype CC; CT/TT, genotype CT/TT. **p < 0.01.

The T allele of SNV rs3093308 was associated with elevated levels of anti-*A. lumbricoides* IgE and parasite antigen induced IL-5 production, both indicators of a strong Th2 response and potential resistance to *A. lumbricoides* infection (45). The presence of one T allele of rs3093308 was associated with higher levels of Th2 cytokines (IL-5 and IL-13, Figures 2A,B) by mitogen-stimulated PBLs among infected individuals (*P* < 0.01). None of the other SNVs studied were associated with alterations in *in vitro* cytokine production by infection status (data not shown). However, *IL21R* gene expression by PBLs was not significantly different between the two alleles (Figure 2C).

### Expression of *WSB1* and *IL21R* in *Ascaris*-Infected and Uninfected Individuals

**Figure 3:**
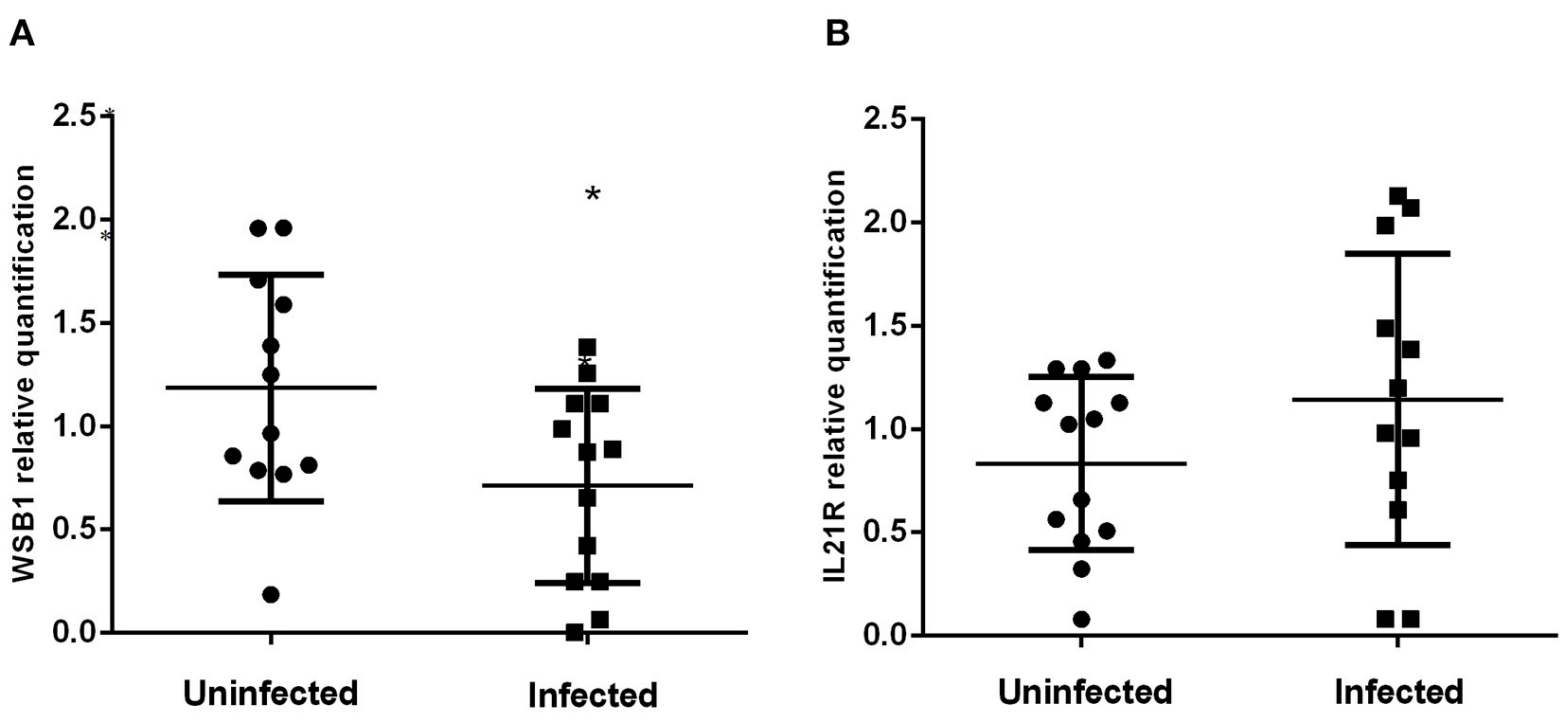
Levels of *WSB1* **(A)** and *IL21R* **(B)** gene expression in *Ascaris lumbricoides*- infected (*N* = 15) and uninfected (*N* = 16) subjects in peripheral blood leukocytes. *P ≤ 0.05.

Figure 3 shows the expression levels of the *WSB1* and *IL21R* genes in *A. lumbricoides*-infected and uninfected individuals. Expression of *WSB1* was lower in infected (*N* = 15) subjects compared to uninfected (*N* = 16) subjects (*P* = 0.0207; Figure 3A). No difference was observed between the two groups for *IL21R* gene expression (Figure 3B).

### Methylation of *WSB1* Promoter Region

**Figure 4:**
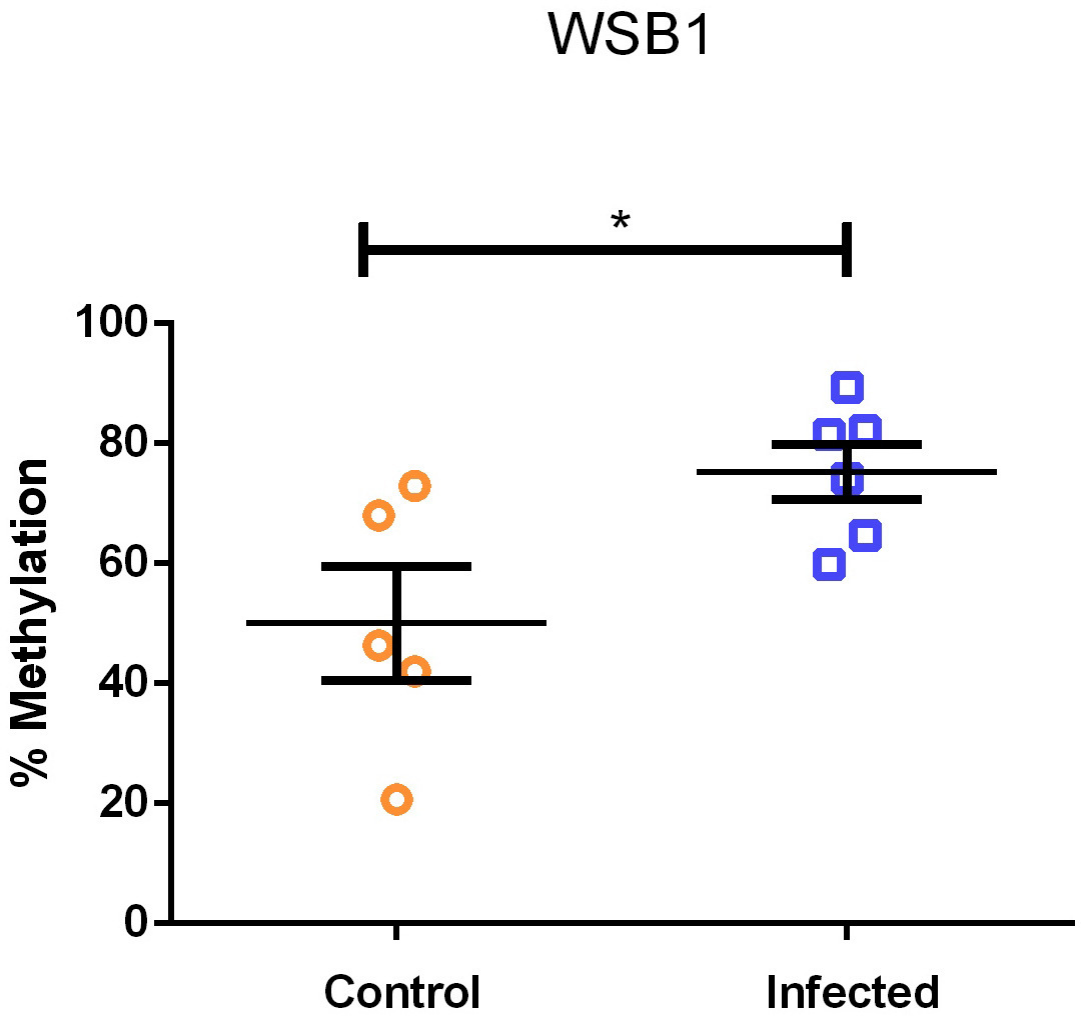
Percentage of DNA methylation in the WSB1 promoter region in *Ascaris lumbricoides*-infected (*N* = 9) and uninfected (*N* = 8) individuals (*P = 0.031, Mann-Whitney-test).

Figure 4 shows the percent methylation of *WSB1* gene in PBLs from *A. lumbricoides*-infected (*N* = 9) and uninfected (*N* = 8) individuals. Infection with *A. lumbricoides* was associated with increased *WSB1* methylation (*P* = 0.031). rs7212516 is in the first intron (position 692 bp). The region included in the methylation analysis is in the promoter region (position −771 bp to −443 bp) containing 14 CpG sites and 4 restriction sites (according to NCBI, Gene ID: 26118). The other SNVs are in the position above 6,000 bp. The amplified region for DNA methylation was analyzed in the reference populations of the 1,000 genome project and found 13 SNVs in that region, two of them with a frequency >1% in Africans and African Americans (https://www.ncbi.nlm.nih.gov/variation/tools/1000genomes/?assm=GCF_000001405.25). These two SNVs do not alter or create restriction sites for the enzymes present in the OneStep qMethyl kit (Zymo Research). In addition, none of the 13 SNVs identified in the 1000 genomes project belong to Illumina BeadChip Human Omni2-8-8 Kit, used here for genotyping as well.

## Discussion

Previous studies suggest that the balance between helminth specific IgE and IgG4 might determine resistance or susceptibility to *helminth* infections, showing that levels of specific IgE have been correlated with resistance to infection, whereas levels of IgG4 have been associated with susceptibility (34–36). In this manuscript, we have conducted two distinct approaches to determine genetic markers associated with *A. lumbricoides* infection. First, we conducted a GWAS for anti-*A*. *lumbricoides* IgE/IgG4 ratio for *A. lumbricoides* infection in a cohort of an admixture population to determine if there are common genetic variants contributing to susceptibility to *A. lumbricoides* infection and as a second phase, based on the GWAS pieces of evidence, we focused our attention to WSB1/*IL21R* pathway, which revealed associations with markers of exposure and cytokine responses to *A. lumbricoides*. For the best of our knowledge, these associations have never been reported before.

We did not identify any novel SNVs meeting genome-wide significance but did identify several SNVs below the genome-wide threshold as being of potential interest: (1) rs7912186 in the *GPR158* gene, described as being linked to plasma membrane scaffold protein in retinal bipolar neurons, contributing to the pathophysiology of steroid-induced ocular hypertension and glaucoma, (46, 47) and also involved in the regulation of the pre-frontal cortex with a potential role chronic stress and depression (48); (2) rs10081726 and rs12550848, located in SMARCA2, that plays a role in the development of lung cancer, hepatocellular carcinoma and esophageal adenocarcinoma (49, 50); and (3) rs7212516, located in the *WSB1* gene on chromosome 17 that is known to be involved in IgE regulation (9).

Previous epidemiological studies have shown inverse associations between levels of anti-*Ascaris* IgE and parasite burden with *A. lumbricoides* indicating a potential role for IgE in resistance to infection (51, 52). WSB-1, a IL-21 receptor binding molecule, enhances the maturation of IL-21 receptor. *WSB1* gene plays an important role in the regulation and maturation of the *IL21R*, and both genes are important for IgE production (9). For this reason, we included the *IL21R* as a gene of biological relevance in our candidate gene analysis. There are several lines of evidence showing that IL-21/IL-21R signaling plays a clear role modulating Type 2 cytokines production (9, 11). Mice deficient for *IL21R* had reduced airways eosinophilia in a model of mite-induced asthma (18), and knock-out mice for *IL21R* expressed higher levels of IgE and lower levels of IgG1 than normal mice after mite antigen exposure (17).

We explored if genetic variants in *WSB1/IL21R* might influence Th2-associated immune responses during *A. lumbricoides* infection using immunological markers of susceptibility and resistance to infection including production of Th2 cytokines *in vitro*. Our results show that variants in these two genes are associated with such markers of the host Th2 response during this helminth infection. SNVs in *WSB1* (rs7213148 and rs8065359) and *IL21R* [rs115350516 (A allele) and rs3093308 (T allele)] were associated with increased production of *A. lumbricoides*-specific IgE (Tables 4, 5) and could be potentially linked to greater resistance to infection. The same two SNVs in *IL21R* SNVs were associated with greater parasite antigen-induced IL-5 production that has been linked to resistance to geohelminth infections (45). These SNVs have not been linked previously to helminth infection or Th2-driven inflammatory conditions.

The T allele of rs3093308 in *IL21R* was associated also with increased production of Th2 cytokines (IL-5 and IL-13) by mitogen-induced PBLs among infected compared to uninfected children (Figures 2A,B), and the same SNV tended to increase *IL21R* gene expression (Figure 2C). These findings could be indicative of a stronger protective immune response against *A. lumbricoides* infection. No previous studies have reported a role for this SNV. In a study evaluating IL21/*IL21R* signaling in murine model of intestinal inflammation, Th2 responses (IL-4 and IL-5 by CD4+ T cells) were markedly suppressed in *IL21R* deficient compared to wild-type mice (53).

Our data can explain, at least in part, findings from previous studies showing elevated *Ascaris*-specific IgE levels to be associated with decreased worm burden and increased resistance to infections with this helminth (51, 52). Other studies have shown significant associations between *locus* 13q33 that includes the genes, LIG4, ABHD13, and TNFSF13B, with *Ascaris*-specific IgE levels (22, 54, 55). Thus, consistent with our findings, genetic regulation of IgE production may play an essential role in susceptibility to *Ascaris* infection.

In our population, the G and T alleles of SNVs rs6505199 and rs9303634, respectively, in *WSB1* (see Table 3), were associated with reduced *Ascaris*-specific IgG4 levels. These results favor increased production of IgE relative to IgG4, the latter known to be a marker of susceptibility to infection (51, 56). Both SNVs were in high linkage disequilibrium (*r*^2^ = 1.00), (see Supplementary Figure 1A). Conversely, the T allele of SNV rs7212516 was positively associated with *Ascaris*-specific IgG4 and perhaps greater susceptibility to the infection.

We also did haplotype analyses in *WSB1* and *IL21R* genes for anti-*A. lumbricoides* IL-5 and IgE production. Two SNVs *IL21R* (rs115350516 and rs3093308) and their haplotypes, especially the AC haplotype, were associated with increased production of IL-5 by *Ascaris*-stimulated PBLs. This same haplotype showed a positive association with anti-*A. lumbricoides* IgE levels. Interestingly, in regression analyses these same SNVs were associated with increased anti-*A. lumbricoides* IgE and IL-5 by *Ascaris*-stimulated PBLs which could be linked to a more effective protective immune response against the parasite. Previous studies have analyzed levels of *WSB1* expression in the brain, spleen, kidney and placenta, primarily with research focusing on cancer development (9, 57). There is no previous study describing the role of *WSB1* in helminth infections or any other Th2-driven condition.

In our gene expression assay, the *WSB1* had lower expression levels in infected subjects when compared with non-infected subjects (Figure 3A). This result allows us to hypothesize that low levels of *WSB1* expression in infected subjects may be related to epigenetic regulation as we have demonstrated increased methylation of the *WSB1* promoter region in infected individuals (Figure 4). Although cell populations within whole blood cultures may differ between cases and controls [infected subjects had greater total leukocyte and eosinophil counts than uninfected subjects (data not shown)], our findings indicated that infection was associated with greater methylation but lower gene expression. However, further studies are required to support a potential effect of *A. lumbricoides* infections on *WSB1* gene hypermethylation. Epigenetic events, such as post-transcriptional modifications of DNA at CPG sites, regulate gene transcription activity, thereby determining the kinetics and final expression (58, 59). On the other hand, there was no statistical difference in gene expression levels for the *IL21R* (Figure 3B). Persistent helminth infections appear to induce changes in DNA methylation in CD4+ cells from helminth-infected individuals. Other epigenetic mechanisms may also be involved in the expression of key genes in the type 2 immune response. A study evaluated histone acetylation in individuals exposed to *A. lumbricoides* found that histone acetylation levels in IL-4 and IL-13 genes were altered by infection (25).

This study has a number of potential limitations including: a relatively small sample size limiting power using the GWAS genome strategy; we were unable to do a replication analysis because of a lack of previous studies collecting data on the same variables (e.g., anti-*A. lumbricoides* IgE or IgG4); and use of whole blood cultures rather than more homogeneous lymphocyte populations due to logistical issues inherent to a population-based study such as ours (26).

Our results, therefore, provide novel mechanistic insights into how helminth infections that affect immune response regulation may modulate also epigenetic processes. Further studies are needed to improve our understanding on how such regulation may occur and the consequences for Th2-driven inflammatory conditions.

## Data Availability Statement

The genetic data sets generated in this study can be found online upon request at https://cidacs.bahia.fiocruz.br/en/platform/epigen-genomic-epidemiology-of-brazilian-cohorts/.

## Ethics Statement

The studies involving human participants were reviewed and approved by Institute of Collective Health, Federal University of Bahia, Salvador, Brazil. Written informed consent to participate in this study was provided by the participants' legal guardian/next of kin.

## Author Contributions

VC, HS, GQ, RV, PO, NC, AP, and RS have conducted the field work, managed the database, and performed the genetic experiments. FS, EB, CM, and RC were responsible for the supervision of the field work and/or laboratory experiments. VC and HS were responsible for data analysis and writing of the manuscript. MB, NA-N, and PC drafting the paper and revising it critically. CF substantial contributions to research design, or the acquisition, analysis or interpretation of data. All authors have actively participated of the analysis of research results and approve the version that is being submitted.

## Conflict of Interest

The authors declare that the research was conducted in the absence of any commercial or financial relationships that could be construed as a potential conflict of interest.
