# Supplementary figures and images for: WSB1 and IL21R Genetic Variants Are Involved in Th2 Immune Responses to Ascaris lumbricoides
Source: Front Immunol. 2021 Feb 22;12:622051. doi: 10.3389/fimmu.2021.622051 (PMC7937724; doi:10.3389/fimmu.2021.622051)

(A)

rs7212516  
rs9303634  
rs8065359  
rs6505199  
rs7213148  
rs9867  
rs1060618

1 5 7 9 10 11 13

11 1 11 0 0 1  
13 13 2 3 43  
2 0 0 5  
0 3 43  
8  
7

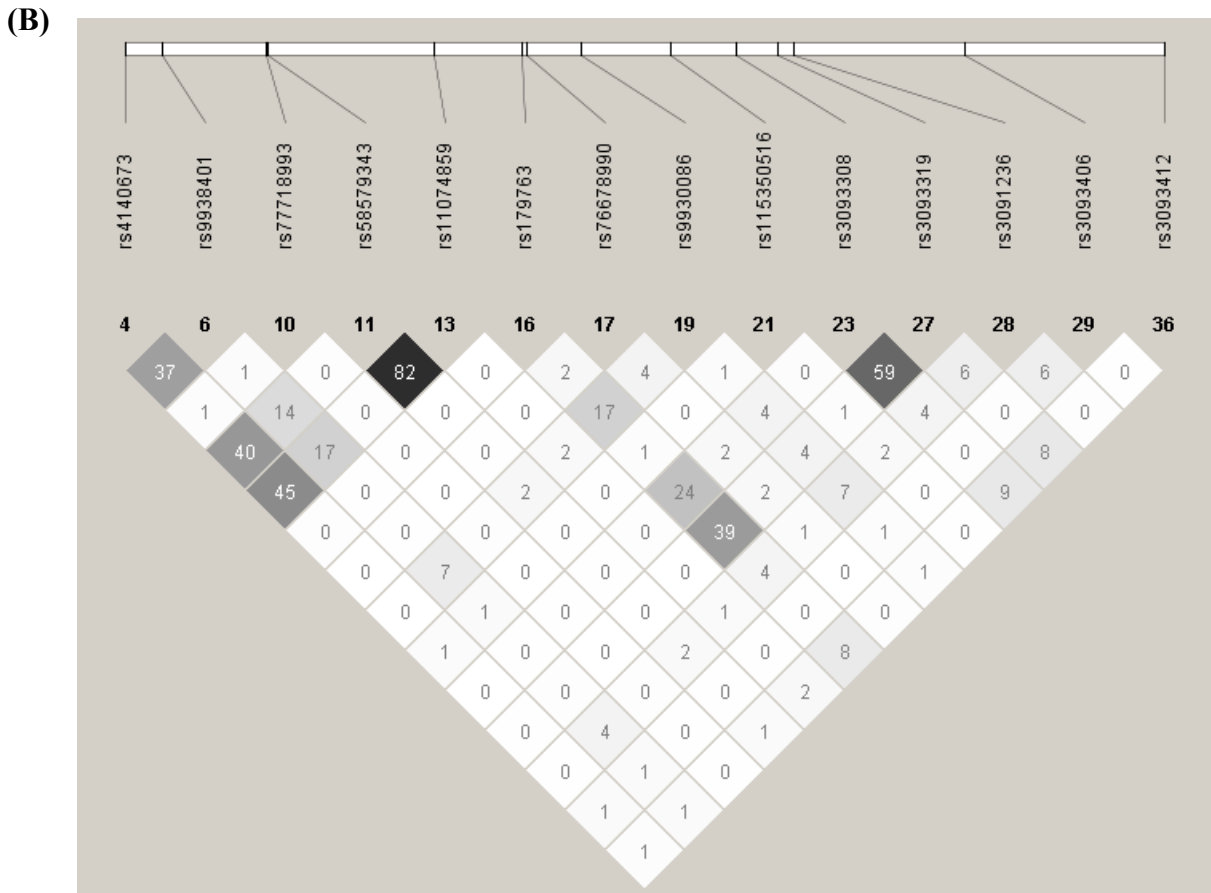

Supplement: Supplementary file 1 [file Image_1.pdf]
