# Supplementary material for: WSB1 and IL21R Genetic Variants Are Involved in Th2 Immune Responses to Ascaris lumbricoides
Source: Front Immunol. 2021 Feb 22;12:622051. doi: 10.3389/fimmu.2021.622051 (PMC7937724; doi:10.3389/fimmu.2021.622051)

Supplementary Figure 2. Quantile-quantile (Q-Q) plot of IgE total and IgG4 specific for Ascaris lumbricoides infection study. Adjusted for sex, age, and the first three principal components for ancestry. Q-Q plot of observed versus expected log10 p values for all tested genotyped single nucleotide variants (SNVs).

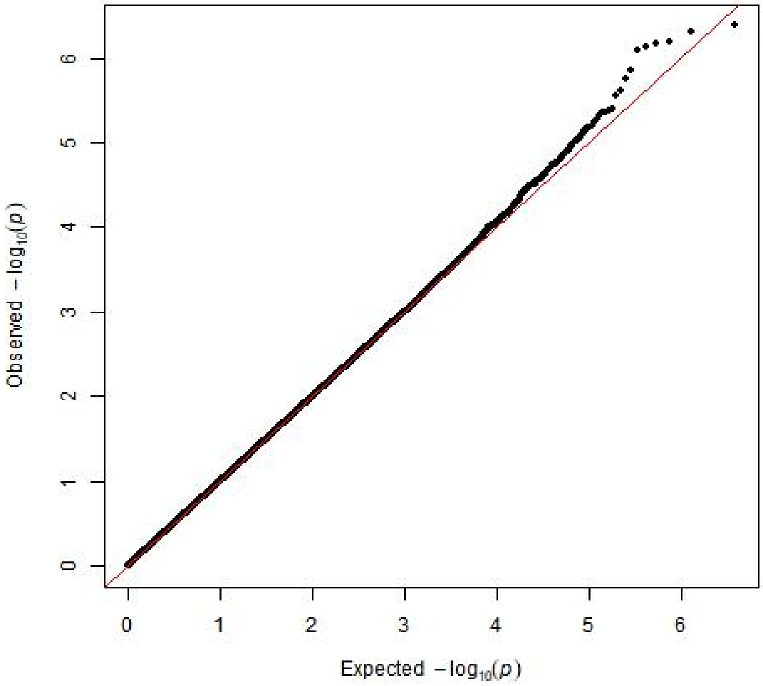

Supplement: Supplementary file 2 [file Image_2.pdf]
