# Supplementary material for: WSB1 and IL21R Genetic Variants Are Involved in Th2 Immune Responses to Ascaris lumbricoides
Source: Front Immunol. 2021 Feb 22;12:622051. doi: 10.3389/fimmu.2021.622051 (PMC7937724; doi:10.3389/fimmu.2021.622051)
Supplement: Supplementary file 3 [file Table_1.DOCX]

| Supplementary Table 1: Annotations of 20 best hits in the GWAs study IgE total and IgG4 for *Ascaris lumbricoides* infection | | | | |
| --- | --- | --- | --- | --- |
| SNV | **Gene** | **Reguloma DB** | **Gene annotation** | **Reference** |
| rs7912186 | GPR158 | 6 | As a novel regulator operating in the prefrontal cortex (PFC) that links chronic stress to depression  Growth and progression in prostate cancer. | [Sutton LP](https://www.ncbi.nlm.nih.gov/pubmed/?term=Sutton%20LP%5BAuthor%5D&cauthor=true&cauthor_uid=29419376), 2018  [Patel N](https://www.ncbi.nlm.nih.gov/pubmed/?term=Patel%20N%5BAuthor%5D&cauthor=true&cauthor_uid=25693195), 2015 |
| rs4782902 | Intergenic | 6 | No Data |  |
| rs200282924 | Intergenic | 5 | No Data |  |
| rs77248612 | LOC105376244 | No Data | No Data |  |
| rs10251182 | Intergenic | No Data | No Data |  |
| rs10081726  rs12550848 | SMARCA2  SMARCA2 | 4  5 | Studies indicate its role in the development of lung cancers, hepatocellular carcinoma and [esophageal adenocarcinoma](https://www.ncbi.nlm.nih.gov/pubmed/28427211/) | Korpanty GJ, 2017; Pasic, 2018; Wu J, 2019 |
| rs4645161 | OSBPL10 | No Data | Associated with dyslipidemia and with peripheral arterial disease | Koriyama H et al, 2010; |
| rs77772209 | Intergenic | 5 | No Data |  |
| rs77284244 | Intergenic | 6 | No Data |  |
| rs12738424 | KAZN | 5 | Regulation of desmosome assembly, embryonic tissue morphogenesis and epidermal differentiation. | [Nachat R](https://www.ncbi.nlm.nih.gov/pubmed/?term=Nachat%20R%5BAuthor%5D&cauthor=true&cauthor_uid=19843585), 2009; [Sevilla LM](https://www.ncbi.nlm.nih.gov/pubmed/?term=Sevilla%20LM%5BAuthor%5D&cauthor=true&cauthor_uid=18840647), 2008. |
| rs7653904 | SORBS2 | 4 | Associated with suppressor of ovarian cancer metastatic colonization | Zhao L, *et al*. 2018. |
| rs1025065 | Intergenic | No Data | No Data |  |
| rs61992474 | Intergenic | 5 | No Data |  |
| rs7219758 | Intergenic | 4 | No Data |  |
| rs11980827 | Intergenic | 6 | No Data |  |
| rs6444926 | Intergenic | No Data | No Data |  |
| rs1998219 | CASC6 | No Data | No Data |  |
| rs7018777 | Intergenic | No Data | No Data |  |
| rs7212516 | WSB1 | 4 | Regulates the metastatic potential in breast cancer.  Plays a role in the regulation and maturation of the interleukin-21 receptor (IL21-R) | Poujade FA, et al. 2018.  [Nara, Onoda *et al.* 2011](#_ENREF_29) |
